# Incorporation of a lambda phage recombination system and EGFP detection to simplify mutagenesis of Herpes simplex virus bacterial artificial chromosomes

**DOI:** 10.1186/1472-6750-7-22

**Published:** 2007-05-14

**Authors:** Falko Schmeisser, Jerry P Weir

**Affiliations:** 1 Laboratory of DNA Viruses, Center for Biologics Evaluation and Research, Food and Drug Administration, Bethesda, MD 20892, USA

## Abstract

**Background:**

Targeted mutagenesis of the herpesvirus genomes has been facilitated by the use of bacterial artificial chromosome (BAC) technology. Such modified genomes have potential uses in understanding viral pathogenesis, gene identification and characterization, and the development of new viral vectors and vaccines. We have previously described the construction of a herpes simplex virus 2 (HSV-2) BAC and the use of an allele replacement strategy to construct HSV-2 recombinants. While the BAC mutagenesis procedure is a powerful method to generate HSV-2 recombinants, particularly in the absence of selective marker in eukaryotic culture, the mutagenesis procedure is still difficult and cumbersome.

**Results:**

Here we describe the incorporation of a phage lambda recombination system into an allele replacement vector. This strategy enables any DNA fragment containing the phage *att*L recombination sites to be efficiently inserted into the *att*R sites of the allele replacement vector using phage lambda clonase. We also describe how the incorporation of *EGFP *into the allele replacement vector can facilitate the selection of the desired cross-over recombinant BACs when the allele replacement reaction is a viral gene deletion. Finally, we incorporate the lambda phage recombination sites directly into an HSV-2 BAC vector for direct recombination of gene cassettes using the phage lambda clonase-driven recombination reaction.

**Conclusion:**

Together, these improvements to the techniques of HSV BAC mutagenesis will facilitate the construction of recombinant herpes simplex viruses and viral vectors.

## Background

Complete viral genomes of large DNA viruses (e.g. herpesviruses) can be incorporated as bacterial artificial chromosomes (BACs). One method is the use of replicons based on the bacterial F plasmid [1]. Since the initial report that described the BAC cloning of a murine cytomegalovirus genome [2], several additional herpesvirus genomes have been cloned as BACs [3-14], including herpes simplex virus 1 (HSV-1) and herpes simplex 2 (HSV-2) [15-18].

BAC technology created new possibilities for the targeted mutagenesis of the herpesvirus genome (for recent reviews, see [19,20]). Since the viral genome can be carried as an episome in bacterial cells, prokaryotic genetic techniques can be employed for manipulation of the viral genome without the need for typical selective mechanisms required for mutagenesis and selection in eukaryotic cells. This allows for the generation of viral mutants that would be difficult to obtain otherwise, either because of the lack of a phenotypic effect due to mutation or because the mutation confers a disadvantage for selection and replication in eukaryotic cells. Viral particles are obtained by transfection of BAC-cloned viral DNA into a permissive cell line. Since the mutant virus is clonally derived, plaque purification of the recombinant virus is usually not needed. In addition, the BAC and BAC-derived virus can be easily shuttled back and forth between *E. coli *and eukaryotic cells, facilitating analysis of the effects of targeted mutation and subsequent sequential mutations. The power and versatility of BAC mutagenesis for studying herpesviruses has been used in numerous ways, including studies on viral pathogenesis, the role of viral antigens in immunity (for examples see [17,21]). Modified viral genomes have potential uses in other areas such as the construction and development of novel viral vectors and vaccines.

Despite the power and the promise of BAC technology, BAC mutagenesis can be difficult and cumbersome. While the generation of a herpes virus BAC is similar in all approaches through introduction of prokaryotic F-factor plasmid features into the viral genome using homologous recombination [2,4], different approaches to BAC mutagenesis have been described. In our past studies, we used the method published by Horsburgh and co-workers [4,15] that uses a gene replacement vector that features a temperature-sensitive origin of replication (*ts-ori*), a positive selection marker (zeocin) and negative selection marker (*sac*B). Other methods to mutagenize herpes BACs such as transposon mutagenesis [22] and PCR-based approaches [23] are efficient and do not require the construction of a special gene replacement vector for each mutation. However, these techniques do not easily allow the generation of certain types of manipulations such as point mutations. BAC recombineering [24,25], a BAC mutagenensis method which uses lambda-mediated homologous recombination in *Escherichia coli*, can be used to efficiently generate extensive mutations in any targeted allele but is an *in vivo *system requiring special *E. coli *strains and is a two step procedure that necessitates that the targeted allele be substituted and then replaced with the desired mutation.

The aim of the present study was to simplify BAC mutagenesis, using approaches tailored to the final application. We believe this method can be used for a broad range of applications. Here we describe two examples how the incorporation of a lambda phage recombination system can greatly simplify the construction of allele replacement vectors. In addition, we describe how EGFP can be utilized for easy screening of positive clones throughout the process of *in vivo *allele replacement in *E. coli *when the allele replacement procedure is a gene deletion. Finally, we describe the incorporation of the lambda phage recombination system directly into an HSV-2 BAC so that gene transfer into a specific viral locus can be performed in a simple one-step *in vitro *reaction. These improvements to the techniques of HSV BAC mutagenesis will facilitate the construction of recombinant herpes simplex virus genomes.

## Results and discussion

### Construction of an improved allele replacement plasmid vector containing lambda phage site specific recombination sites

**Figure 1:**
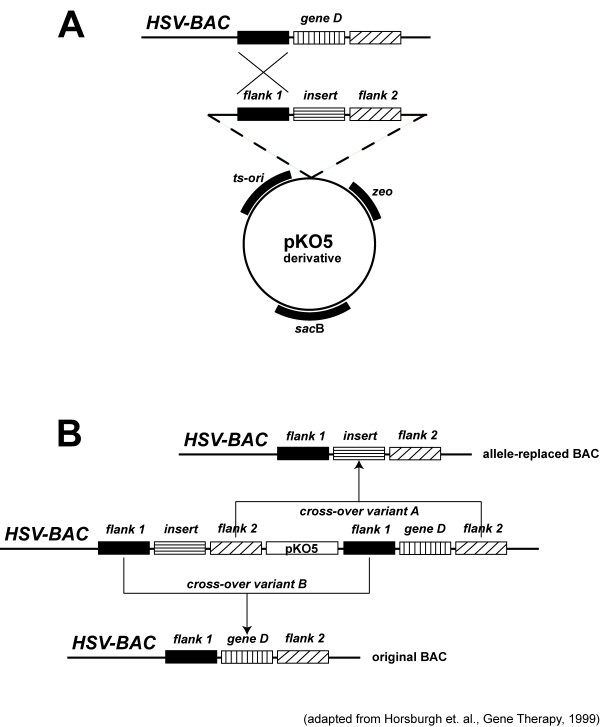
**Schematic diagram of allele replacement strategy using vector pKO5**. (A) Features of a typical allele replacement vector include a positive selection marker for antibiotic resistance marker (*zeo*), a temperature-sensitive origin of replication (*ts-ori*), a negative selection marker (*sac*B) and flanking sequences to targeting cross-over at hypothetical gene D. Incubation at non-permissive temperature (43°C) and selection on Zeocin/Chloramphenicol lead to co-integration through homologous recombination (single cross-over). (B) Diagram of the resultant single-crossover recombinant BAC and the two possible forms following resolution. Incubation at permissive temperature (30°C) and selection on Zeocin/Sucrose lead to resolution of the co-integrate, excision of the plasmid through homologous recombination (double cross-over).

An allele replacement strategy for mutagenesis of bacterial artificial chromosomes has been previously described [4]. This strategy utilizes plasmid replacement vector pKO5, containing the elements for positive selection of the integrated allele via a single cross-over event (a temperature-sensitive origin of replication *ts-ori*, and a zeocin resistance gene *zeo*), and an element for negative selection to force recombination necessary for allele replacement (*sac*B). A simplified version of the allele replacement strategy is shown in Figure 1.

**Figure 2:**
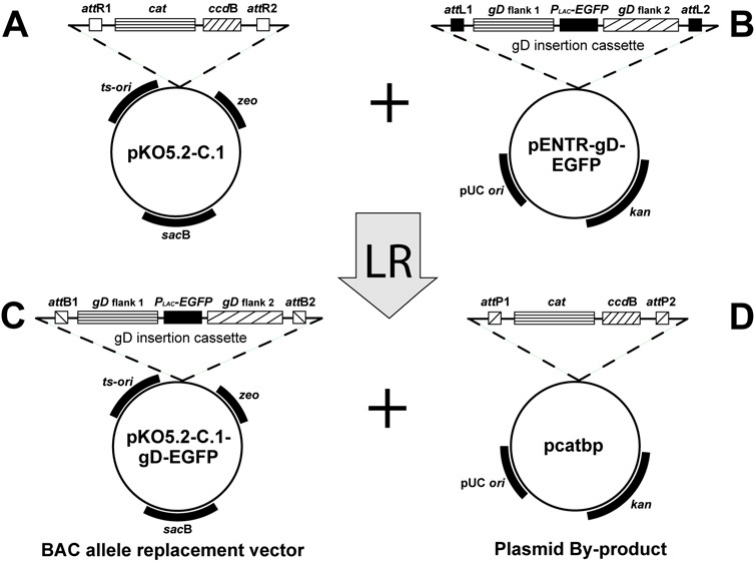
**Construction of allele replacement vector pKO5.2-C.1-gD-EGFP using LR recombination**. (A) Schematic diagram of the pKO5.2-C.1 plasmid vector containing the original KO5 selection markers, the lambda recombination sites (*att*R) and the negative selection marker for recombination (*ccd*B gene). (B) Schematic diagram of the plasmid pENTR-gD-EGFP containing *att*L recombination sites and the *EGFP *gene in place of the HSV-2 glycoprotein D gene. (C) Schematic diagram of the resulting allele insertion vector following *in vitro *recombination between pKO5.2-C.1 and pENTR-gD-EGFP. (D) Schematic diagram of the resulting plasmid by-product of the LR recombination reaction.

Plasmids such as pKO5 contain positive and negative selection markers making it extremely powerful for BAC mutagenesis. However, plasmids derived from pKO5 are difficult cloning vectors to use because of the limited number of restriction sites available for insertion and the low frequency of recombinant transformants obtained during the cloning procedure. As a means to address these limitations we inserted an ~1.6 kb DNA fragment containing *att*R lambda phage recombination sites into pKO5. A schematic drawing of the constructed vector pKO5.2-C.1 is shown in Figure 2. Phage lambda recombination is a highly specific, directional, and conservative recombination reaction, mediated by the lambda clonase enzyme, that occurs between two site-specific attachment sites (*att*L and *att*R). In addition to the lambda recombination sites, this gene cassette contains the *ccd*B gene [26] which is a negative selection marker designed to minimize the number of non-recombinant transformants obtained during cloning. Thus, any DNA fragment containing *att*L recombination sites can be efficiently inserted into the *att*R sites in the pKO5.2-C.1 vector using phage lambda clonase (designated as the LR recombination reaction), and the resultant recombinant plasmids are ready for the allele replacement reaction.

### Use of EGFP to facilitate screening of BACs containing replaced alleles

As depicted in Figure 1, the allele replacement procedure includes a single cross over event that results in the generation of a BAC intermediate that contains two copies of the targeted allele, one from the starting BAC and one from the replacement vector. In theory, resolution of this intermediate should have an equal chance of generating the original BAC or a new BAC with the replaced allele. In practice, we have not found this to be the case, with the percentage of screened clones containing the replaced allele as low as 5%, but always much lower than the expected 50% (data not shown). For example, in a previously reported study, we constructed an allele replacement vector using the improved vector pKO5.2-C.1 as described above, and used BAC mutagenesis to generate an HSV-2 genome that was deleted for the essential gene encoding glycoprotein D [17]. While the construction of the allele replacement vector containing the flanking regions of the gD gene, but missing the coding sequences, was relatively straight-forward, a large number of colonies in the final step of the allele replacement procedure had to be screened in order to find the desired BAC recombinant with the gD deletion. It is likely that the percentage of replaced allele to starting allele containing BACs is at least somewhat dependent upon the size of the flanking sequences used in the allele replacement vector, but construction of allele replacement vectors with exceptionally large flanking sequences is not always practical. Consequently, we reasoned that for allele replacement reactions which are gene deletions, co-expression of EGFP in the replacement vector would aid in the detection of the desired cross-over recombinant BACs.

**Figure 3:**
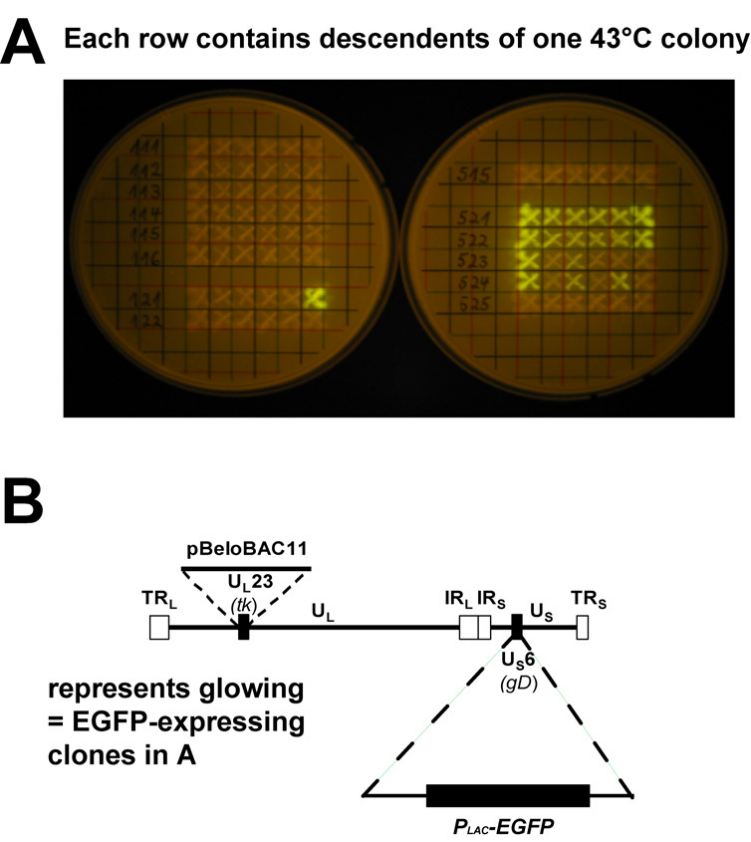
**Construction of HSV2-BAC ΔgD**. (A) Visualization of EGFP-containing BAC colonies after transformation following the allele replacement procedure. Bacterial clones harboring mutant (*ΔgD*) HSV2-BAC glow green because of insertion of *EGFP *at the site of *US6*. (B) Schematic diagram of HSV2-BAC with *EGFP *insertion at *US6*(*gD*). pBeloBAC11 refers to the insertion of the BAC sequences into *UL23 *(*tk*) of the viral genome.

To evaluate the feasibility of EGFP co-detection for obtaining the desired resolved BAC containing a targeted gene deletion, we designed a new HSV-2 gD-deletion replacement vector containing the *EGFP *gene in place of the *gD *coding sequences and transferred this chimeric gene to the BAC insertion shuttle vector pKO5.2-C.1 by the LR recombination reaction, resulting in pKO5.2-C.1-gD-EGFP (Figure 2). The gD flanks with the inserted EGFP were then transferred into the HSV-2 BAC by transformation of HSV2-BAC-containing RR1 electrocompetent cells and selection of bacterial colonies on chloramphenicol/zeocin (Cam/Zeo) plates at 30°C. Colonies at this stage contained both the HSV2-BAC (choramphenicol marker) and the allele insertion plasmid (zeocin marker) but not necessarily a recombinant BAC because both the BAC and the plasmid will replicate at this temperature. The following day, multiple colonies were picked into 1 ml of LB broth and plated in serial dilutions onto Cam/Zeo plates at 43°C, conditions that require integration of the insertion plasmid into the HSV2-BAC vector. Individual colonies from the 43°C plate were re-plated onto sucrose/chloramphenicol (Suc/Cam) plates at 30°C to force resolution of the single cross-over co-integrate. Six colonies, selected from each of 14 original 43°C colonies, were then transferred to chloramphenicol plates at 30°C and visualized for EGFP expression (Figure 3A). Five of the 14 colonies we selected as a representative sample expressed the *EGFP *indicator gene, and were shown by PCR analysis to contain the *EGFP *gene replacing the *gD *gene (data not shown). In addition, as previously described [17], virus derived from the *gD*-deleted/*EGFP*-inserted HSV2-BAC was unable to grow on Vero cells following transfection, whereas viable virus was recovered following transfection of VD60 cells (Vero-derived cells expressing glycoprotein D). Eukaryotic cells infected with the resulting recombinant virus did not express EGFP, consistent with the fact that the inserted *EGFP *gene is under control of a prokaryotic promoter. A schematic diagram of the BAC-derived HSV-2 genome containing *EGFP *inserted into the *gD *gene is shown in Figure 3B. Thus, expression of EGFP is a suitable choice for screening of the BAC recombinant containing insertions, especially when the percentage of allele replaced BACs is relatively low.

### Use of lambda phage site specific recombination sites in an HSV-2 BAC vector for direct recombination of gene cassettes using the LR recombination reaction

Although both procedures described above simplify certain aspects of the allele replacement reaction, the process still requires the construction of a plasmid replacement vector and is a multi-step selection procedure. Consequently, we were interested in determining whether gene replacements could be performed directly in the BAC using the efficient lambda phage recombination system.

**Figure 4:**
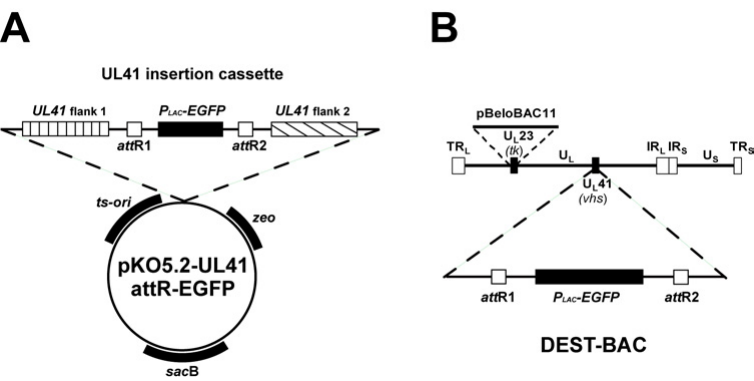
**Construction of an HSV-2 BAC containing *att*R lambda recombination sites**. (A) Schematic diagram of allele replacement vector pKO5.2-UL41-attR-EGFP containing *UL41 *sequences flanking the *att*R sites on either side of the *EGFP *gene cassette. (B) Schematic diagram of DEST-BAC, and HSV2-BAC containing the *EGFP *gene flanked by lambda phage *att*R sites for targeting gene replacements into *UL41*.

We chose the *UL41 *gene for insertion of *att*R recombination sites into the HSV2-BAC since it is known that the *UL41 *gene encodes the virion host shutoff (vhs) protein that is dispensable for virus replication in cell culture. Toward this end, an insertion plasmid was constructed that contained ~4 kb of DNA sequences surrounding the *UL41 *gene. In place of the *UL41 *ORF, we inserted the gene encoding EGFP under control of a prokaryotic promoter flanked by the *att*R recombination sites. A schematic diagram of the pKO5.2-UL41 attR-EGFP is shown in Figure 4A. This insertion vector also contained the previously described selection markers, the *att*R recombination sites, and the *EGFP *gene. Screening of the recombinant HSV-BAC containing *att*R-*EGFP *in *UL41 *was aided by expression of the EGFP in bacterial colonies and insertion was confirmed by PCR analysis using primer pairs in the *EGFP *gene and the flanking *UL41 *sequences (data not shown). The resulting HSV-BAC, designated as DEST-BAC (Figure 4B), was isolated to study its suitability as an *in vitro *destination vector for DNA fragments flanked by *att*L sequences. This BAC DNA was infectious when transfected into Vero cells (data not shown).

**Figure 5:**
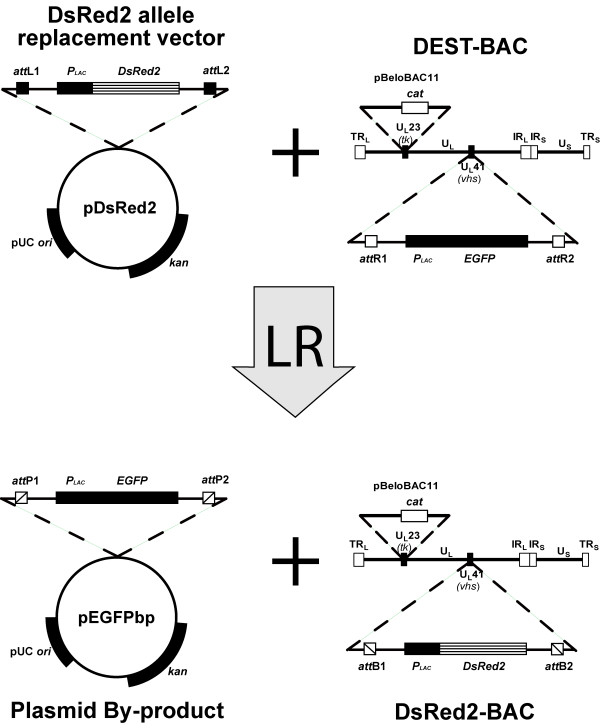
**Outline of the recombination reaction between DsRed2 allele replacement vector and DEST-BAC**. Products of the forward reaction are the recombinant BAC (DsRed2-BAC) and a plasmid by-product (pEGFPbp).

**Figure 6:**
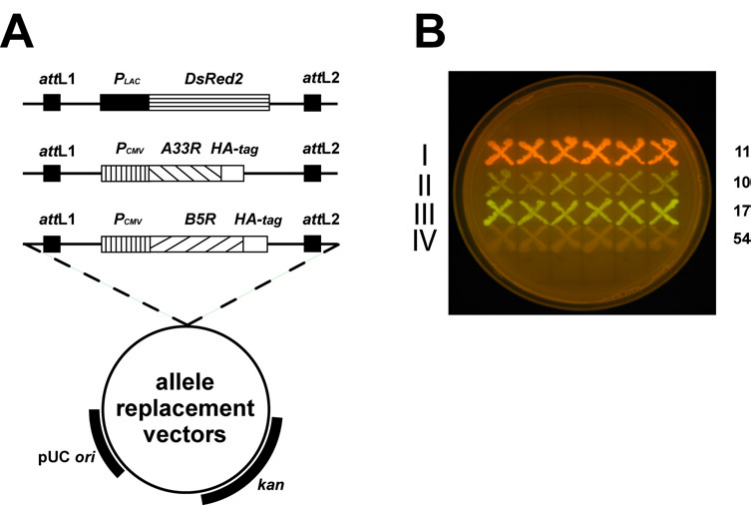
***In vitro *recombination of allele replacement vectors into DEST-BAC, transformation, and visualization of resultant phenotypes**. (A) Schematic diagram of allele replacement vectors featuring *DsRed2*, *A33R*, and *B5R*. (B) Four visual phenotypes of colony transformants observed after *in vitro *recombination between DEST-BAC and pDsRed2 and transformation. Colony transformants from a typical transformation were replated onto a chloramphenicol-containing agar plate to demonstrate phenotype differences. Numbers to the right of the plate refer to the actual numbers of each colony phenotype observed on the original transformation plate.

Unlike the allele plasmid replacement vectors containing *att*R recombination sites (Figure 2A), the DEST-BAC does not contain a negative selection marker such as the *ccd*B gene. Thus, following an *in vitro *recombination reaction with DEST-BAC and a fragment containing the *att*L sites, there is no suppression of the growth of bacterial transformants harboring either the original HSV-BAC and/or the plasmid by-products of the LR reaction (Figure 5). Nevertheless, DEST-BAC contains the *EGFP *gene which is expressed in prokaryotic cells and thus screening of recombinant BACs into which insertion has occurred should be facilitated by loss of this marker. In order to evaluate direct recombination into DEST-BAC via the *in vitro *recombination reaction, we constructed several vectors, each containing a different DNA fragment between *att*L recombination sites (Figure 6A). One of the plasmid vectors contained the gene encoding DsRed2 under control of a prokaryotic promoter to allow visualization of the various BAC recombinants that might be generated following the recombination reaction. The other two vectors contained the genes encoding the vaccinia virus A33R and B5R glycoproteins under control of eukaryotic promoters to determine the ability of inserted genes to be expressed in the recombinant BAC viruses. The *A33R *and *B5R *genes were each fused to an epitope tag from the influenza hemagglutinin gene for easy identification of the expressed gene product.

Recombination between the high copy number pDsRed2 vector and the low copy number GFP-expressing DEST-BAC was carried out in the standard *in vitro *reaction, followed by transformation of electrocompetent *E. coli *DH10B and plating onto LB plates containing either Chloramphenicol or Kanamycin. Transformants arising on Cam plates derived from the reaction with pDsRed2 (Figure 6A) showed four distinct phenotypes, bright red (I), weak green (II), bright green (III), and not glowing/weakly red (IV) (Figure 6B). The majority (54 of 92) of transformants were of the not glowing/weakly red (IV) phenotype (numbers on the right in Fig. 6B). These colonies were able to grow in the presence of chloramphenicol, but not kanamycin, suggesting the presence of the recombinant DsRed2-inserted BAC. Transformants of the weak green (II) phenotype were also able to grow in the presence of chloramphenicol but not kanamycin, suggesting the presence of the starting EGFP-expressing BAC. In contrast, the bright red colonies (row I) and the bright green colonies (row III) were also able to grow on LB Kan plates (data not shown), suggesting that these types of colonies contained a kanamycin resistant plasmid (either the plasmid by-product of the *in vitro *recombination reaction or the original vector) in addition to a chloramphenicol-resistant BAC. Further studies demonstrated that by reducing the concentration of DNA used for transformation, the number of such double transformants could be reduced and also result in higher overall transformation efficiency (data not shown).

**Figure 7:**
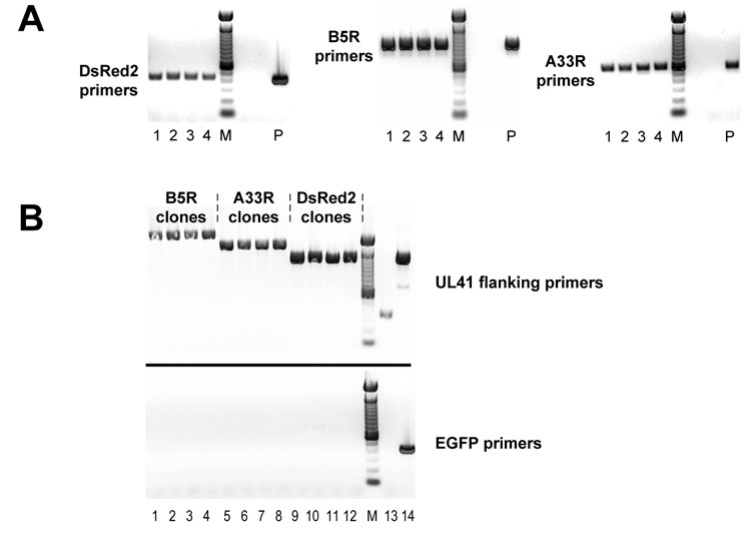
**PCR Analysis of HSV-2 BAC recombinants**. (A) Insert-specific PCR of 4 individual clones each of HSV2-BAC recombinants containing an inserted *DsRed2 *(left panel), vaccinia virus *B5R *(middle panel) or vaccinia virus *A33R *gene (right panel). Lane M -100 bp molecular size markers. Lane P – Specific plasmid controls containing the genes for DsRed2, B5R, A33R, respectively (from left to right). Sizes of PCR products are: DsRed2 – 424 bp; B5R – 1014 bp; A33R – 586 bp. (B) *UL41 *and *EGFP *PCR analysis of 4 individual clones each of HSV2-BAC recombinants containing an inserted vaccinia virus *B5R *(lanes 1–4), vaccinia virus *A33R *gene (lanes 5–7), or *DsRed2 *gene (lanes 9–12). Lane M -100 bp molecular size markers. Lane 13 – HSV2-BAC. Lane 14 – DEST-BAC. The top half of the gel shows the PCR products generated with UL41 primers for each clone. Sizes of UL41 PCR products are: B5R – 2280 bp; A33R – 1850 bp; DsRed2 – 1290 bp (DsRed2); HSV2-BAC – 342 bp; UL41 PCR of DEST-BAC – 1320 bp). The bottom half of the gel shows the PCR products using EGFP primers for each clone, confirming the absence of the EGFP in each recombinant. Size of EGFP PCR product of DEST-BAC is 433 bp (lane 14).

The color and antibiotic sensitivity results suggested that the desired HSV BAC containing the *DsRed2 *insert would be contained in colonies with the not glowing/weakly emitting red phenotype due to the fact that the DsRed2 indicator gene product is only barely visible in colonies. This phenotype, compared to the brighter green appearance of colonies expressing EGFP protein, is presumably because of the low BAC copy number and the slower maturation of the functional tetrameric DsRed2 protein compared to the monomeric EGFP protein. To determine whether the weakly emitting red colonies contained the desired HSV BAC, individual colonies of this phenotype were analyzed by PCR using primer sets specific for *DsRed2*, the *UL41 *flanking sequences, *EGFP*, and 3 HSV-2 genes well separated on the HSV-2 genome (*UL2, UL45*, and *US12*). Figure 7A (left panel) shows the *DsRed2 *PCR results for 4 of the colonies. The presence of the 424 bp product from the PCR using the DsRed2 primer set indicates that each colony contained the *DsRed2 *gene. These same colonies were negative for *EGFP *by PCR using the primer set specific for that gene (bottom half of Figure 7B). In addition, the PCR using the UL41 primer set generated the expected 1290 bp product consistent with elimination of the 970 bp containing the *EGFP *gene from DEST-BAC and insertion of the 940 bp containing the *DsRed2 *gene (top half of Figure 7B). Finally, the presence of HSV-2 viral sequences in each clone was confirmed with the PCR using the HSV-2 primer sets (data not shown). Together, these results indicated that the pale glowing/weakly emitting red colonies contained the HSV BAC with the inserted *DsRed2 *gene. The recombinant BAC DNA was infectious as shown by formation of virus after transfection of the BAC DNA from 2 independent clones into Vero cells (data not shown). An additional 12 colonies, selected from the plate on the basis of weakly emitting red phenotype were analyzed in the same manner, and the results were identical to those of the first 4 colonies shown in Figure 7 (data not shown). Therefore, direct recombination into the HSV-BAC template is feasible for BAC insertion mutagenesis, and that the lack of a negative selection (e.g., *ccd*B gene) was not critical for the selection procedure. Since colonies without color yielded the desired HSV BAC, these results suggest that this technique would be applicable for the insertion of any fragment flanked by *att*L recombination sites, particularly if the starting BAC template expressed an *EGFP *gene.

Similarly, we investigated the feasibility of direct *in vitro *recombination into DEST-BAC using the *att*L flanking *B5R *and *A33R *gene constructions (schematic diagrams shown in Figure 6A). Since there was no marker gene for visualization of the desired recombinant BAC, transformants showed only three phenotypes on LB Cam plates, bright green, slightly green, and non-glowing. As in the DsRed2 example, very bright green colonies were viable on LB Kan plates suggesting the presence of an *EGFP*-containing plasmid by-product in addition to a BAC. Since slightly green colonies growing on chloramphenicol suggested the presence of the starting BAC template, we analyzed colonies without color and that were unable to grow on LB Kan plates for the presence of the desired *B5R *or *A33R *BAC recombinant. Four clones each from the B5R and A33R recombination reaction/transformation were screened for the presence of the transgene by PCR using sequence-specific primers for *B5R *or *A33R *(Figure 7A). Each colony without color from the B5R reaction had the expected 1014 bp B5R PCR product (Figure 7A, middle panel) and each colony without color from the A33R reaction had the expected 586 bp A33R PCR product (Figure 7A, right panel). Absence of a PCR DNA fragment when using *EGFP *primers confirmed the loss of the *EGFP *gene in all of these colonies (bottom half of Figure 7B). In addition, the PCR using the UL41 primer set revealed the expected sizes at the UL41 locus for both the B5R and A33R recombinant HSV-BACs, 2280 bp and 1850 bp, respectively (top half of Figure 7B).

**Figure 8:**
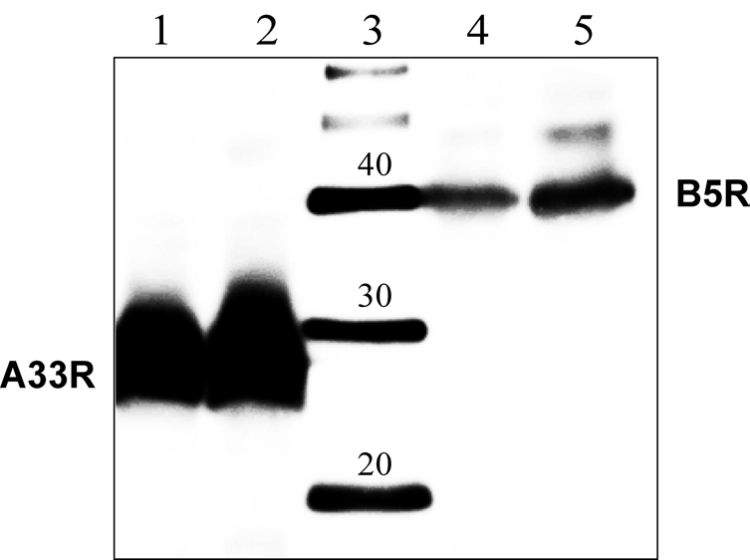
**Expression of transgenes from recombinant HSV-2 BACs**. Vero cells were infected with either A33R-expressing (Lanes 1–2) or B5R-expressing (Lanes 4–5) HSV2-BAC recombinant viruses. Infected cells were harvested and analyzed by Western blot using mouse anti-HA monoclonal antibody. Detection was done with an HRP-conjugated sheep anti-mouse antibody. The blot was visualized by chemiluminescence. Lane 3 – Molecular size markers (kDa).

To determine whether the recombinant *A33R*- and *B5R*-containing HSV BACs expressed the inserted proteins from the *UL41 *locus, Western blot analysis was performed. Infectious virus derived from BAC-transfected Vero cells was used to prepare a virus stock for subsequent infection experiments. Lysates from infected Vero cells were harvested and the proteins separated on a polyacrylamide gel and transferred to nitrocellulose membranes for immunoblotting with a monoclonal antibody against the HA epitope tag that is present on both recombinant proteins (Figure 8). In each case, an expressed protein of the predicted size was apparent.

**Figure 9:**
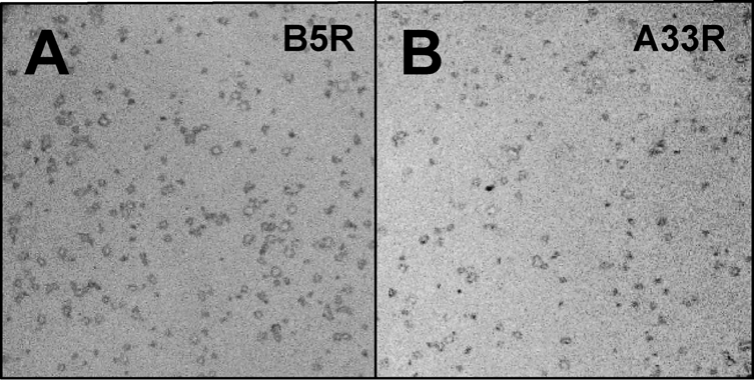
**Immunostained viral plaques of recombinant viruses derived by the BAC allele replacement technique**. Virus was isolated following transfection of Vero cells with recombinant HSV2-BACs expressing A33R or B5R, titered and used to infect plates of Vero cells. Monolayers were fixed and then probed with mouse anti-HA monoclonal antibody, followed by AP-conjugated goat anti-mouse antibody. (A) B5R-expressing HSV2-BAC virus infected cells. (B) A33R-expressing HSV2-BAC virus infected cells.

Recombinant viruses isolated through the BAC mutagenesis procedure should be clonal, thus eliminating the need for further plaque purification following transfection of the BAC DNA into eukaryotic cells. To confirm this, we infected Vero cell cultures with the A33R- and B5R-expressing recombinant viruses obtained from the BAC insertion procedure. After plaques started to appear, cell monolayers were fixed and probed with the mouse anti-HA monoclonal antibody (Figure 9). In plates containing hundreds of virus plaques, every plaque was stained by the monoclonal to the epitope tag, indicating both the clonal nature of the virus derived by BAC mutagenesis as well as the stability of the derived recombinant viruses.

We have shown that any DNA fragment flanked by *att*L recombination sites can be inserted directly into DEST-BAC or any other HSV BAC containing *att*R sites. This procedure is feasible even without a negative selection marker such as the *ccd*B gene. While there is no selective pressure against bacterial colonies harboring the original DEST-BAC, the majority of colony transformants obtained harbor the desired recombinant BAC (Fig. 6B). This is likely due to the molar ratio of *att*R sites to *att*L sites in the *in vitro *recombination reaction. Since the *att*R-containing BAC is approximately 50 times as large as the *att*L-containing replacement vector, there will be a limiting number of *att*R sites and the *in vitro *recombination reaction will be driven towards insertion of the fragment from the allele replacement plasmid into the BAC.

Somewhat similar recombinase systems have recently been reported that describe the construction of recombinant herpesvirus BACs with inserts into ICP6 rather than UL41 [27,28]. In these systems, the Cre/lox recombinase system was used to insert a transgene into the ICP6 gene of HSV, the same site that was engineered to harbor the BAC replication sequences. A second recombination system (FLPe/FRT) was used during transfection of mammalian cells to eliminate the BAC sequences. The desired recombinant, missing the BAC sequence but with the desired insert at the ICP6 locus, was then plaque purified. These systems have the advantage of being able to yield a recombinant virus with an inserted transgene without remaining prokaryotic BAC sequences. The procedure however, requiring more manipulations than the DEST-BAC procedure and plaque purification of the final virus product, is relatively lengthy. Thus, each system has its advantages, and the most appropriate system to be used for construction of a recombinant herpes virus using a BAC system will depend upon the desired characteristics of the recombinant virus that is being constructed.

## Conclusion

In summary, BAC technology is an extremely powerful method for construction of complex recombinant herpes simplex viruses, particularly in instances when there is no selective mechanism for the desired recombinant. The improvements to the allele replacement procedure described in this report facilitate both complex and routine constructions. For any type of allele replacement, cloning into pKO5-derived vectors is greatly simplified by use of the lambda recombination system. The clonase enzymes are commercially available and provide high lot-to-lot consistency for reliable results. In addition, there are a large number of vectors available that incorporate the *att*L recombination sites making it easy to integrate BAC mutagenesis with projects involving expression studies of target proteins in different expression systems. For allele replacements which are gene deletions, selection of bacterial colonies containing the desired BAC-deletion mutant is aided by the use of EGFP visualization. Finally, construction of series of viruses or vectors containing mutations or insertions into a single locus of the viral genome can be expedited by construction of a recipient BAC containing the lambda recombination sites followed by direct recombination into the HSV-BAC. Taken together, these techniques provide improved methods for the construction of recombinant herpes simplex viruses and virus vectors.

## Methods

### Construction of plasmids and BACs

Platinum Pfx DNA polymerase (Invitrogen, Carlsbad, CA) was used for PCR reactions to amplify DNA sequences for subsequent cloning. Takara ExTaq polymerase (Takara, New York, NY) was used for all analytical PCR. The authenticity of all constructs throughout this study was confirmed by sequencing using the BigDye Terminator v3.1 Cycle Sequencing Kit (Applied Biosystems, Foster City, CA). Plasmid DNA was isolated using Qiawell or Qiaprep Plasmid prep (Qiagen Inc., Valencia, CA). Electrocompetent RR1 cells were prepared using ice-cold water and 10% glycerol essentially as described in [29]. BAC DNA was isolated from a large culture (1.5 liters) using Qiagen's Large-Construct kit. Routine molecular biology procedures used in the course of plasmid constructions and characterization were based on those described in [29]. Restriction enzymes, calf intestinal phosphatase (CIP), and Klenow fragment used to blunt DNA fragments were obtained from NEB (Beverly, MA).

Antibiotics and supplements in solid agar plates and liquid media were used at the following concentrations: zeocin (Zeo), 25 μg/ml; kanamycin (Kan), 50 μg/ml; chloramphenicol (Cam), 20 μg/ml; Ampicillin (Amp), 100 μg/ml; Sucrose (Suc) 5% (w/v).

### pKO5.2-C.1

Vector pKO5.2-C.1 is a derivative of pKO5, modified for use as a destination vector by the insertion of a gene cassette containing *att*R recombination sites into the multiple cloning region. To achieve that, pKO5 was digested with *Sal*I and *Not*I and treated with calf intestinal phosphatase (CIP). Reading frame cassette C.1 (Gateway vector conversion system, Invitrogen) was ligated with the linearized pKO5 and transformed into *E. coli *DB3.1 cells (Invitrogen). DB3.1 cells are resistant to the effects of the product of *ccd*B gene located between the *att*R flanks of C.1 reading frame in pKO5.2-C.1.

### pENTR-gD-EGFP

This vector is based on a previously described plasmid pENTR-1A-gD5678 [17]. To construct pENTR-gD-EGFP, pEGFP (Clontech, Mountain View, CA) was digested with *Pvu*II and *Stu*I to obtain an ~1 kb fragment harboring the gene coding for the Enhanced Green Fluorescent Protein (*EGFP*) downstream of a prokaryotic (*Plac*) promoter. The fragment was cloned into a *Kpn*I-digested, Klenow-blunted pENTR-1A-gD5678, to obtain pENTR-gD-EGFP. The *gD *flanks with the inserted *EGFP *were transferred by LR recombination to the BAC insertion shuttle vector pKO5.2-C.1, to generate pKO5.2-C.1-gD-EGFP.

### DEST-BAC

To construct the *UL41*-insertion vector, two regions of the HSV-2 (Genbank accession number NC_001798) *UL40-UL42 *region were amplified by PCR using the following primers:

UL41_BglII (5'GCG**AGATCT**GTATGTCACAGAGAAGGCGGACGGG)

UL41_EcoRI (5'GCG**GAATTC**TGGCCCAACAGATCGCGGGCGAGG)

UL41_HindIII (5'GCG**AAGCTT**GAAGTACTGCAGCAGGTCGCGGCAG)

UL41_XhoI (5'GCG**CTCGAG**AATGTGCGGTGTGTGTTTTCCCCG)

The resulting *Bgl*II-*Eco*RI and *Hin*dIII-*Xho*I fragments containing approximately 2 kb downstream and upstream flanking sequences of the *UL41 *gene, respectively, were cloned into pSP72 (Promega, Madison, WI) to generate pUL41. pUL41 was digested with enzymes *Hin*dIII and *Eco*RI and treated with Klenow. Reading frame cassette A (Gateway vector conversion system, Invitrogen) was ligated into the linearized vector, giving rise to plasmid vector pUL41-attR-ccdB-cat. The plasmid was maintained in *E. coli *DB3.1 cells.

Plasmid pUL41-attR-ccdB-cat was digested with *Sal*I, treated with Klenow, and was partially digested with *Not*I. The product was ligated with a *Pvu*II/*Not*I fragment from plasmid pEGFP, containing the *EGFP *gene with a prokaryotic promoter (*Plac*). The resulting construct, pUL41-attR-EGFP, was digested with *Bgl*II/*Xho*I. A fragment containing the *UL41 *flanks with *att*R sequences and *EGFP *was transferred to *Bgl*II/*Xho*I digested pKO5.2, to obtain pKO5.2-UL41-attR-EGFP, the shuttle vector for allele replacement into *UL41 *locus of HSV2-BAC. DEST-BAC was generated from HSV2-BAC by allele replacement of the HSV2-BAC *UL41 *gene with the disrupted *UL41 *gene containing the *att*R flanks and *EGFP*. Successful allele replacement was detected visually by expression of EGFP, and confirmed by PCR analysis, using three sets of HSV2-specific primer pairs, *UL41 *primers flanking the insertion site, and primers amplifying a product within *EGFP *gene. The resulting recombinant BAC DNA was transfected into Vero cells. Presence of plaques on a Vero cell monolayer confirmed that the recombinant BAC is infectious.

### *DsRed2*, *A33R*, and *B5R *allele replacement vectors

A set of allele replacement vectors were designed to study aspects of the LR recombination reaction and to investigate transfer of a suitable transgene to a viral BAC. These vectors are based on pENTR-1A (Invitrogen) and contained either *DsRed2 *or the vaccinia virus *A33R *or *B5R *genes. To insert the *DsRed2 *gene, pENTR-1A was digested with *Sal*I/*Not*I, followed by treatment with Klenow to fill in overhangs, and ligation of the *Pvu*II/*Stu*I fragment containing *DsRed2 *and the *lac *promoter (pDsRed2, Clontech). To insert the *B5R *gene, plasmid pDNA3-B5R-HA (Clement Meseda, manuscript in preparation) was digested with restriction enzymes *Bgl*II (blunted with Klenow) and *Pvu*II to obtain an expression cassette of vaccinia virus *B5R *gene fused to an HA-tag under the control of the CMV promoter. The fragment was than cloned into the blunted, *Sal*I/*Not*I digested pENTR-1A vector. Similarly, the plasmid pcDNA3-A33R-HA (Clement Meseda, manuscript in preparation) was digested with restriction enzymes *Nru*I and *Pvu*II, to obtain an expression cassette of the *A33R *gene with an HA-tag under the control of the CMV promoter, and ligated with *Sal*I/*Not*I digested, Klenow-treated pENTR-1A vector. Transformed *E. coli *harboring entry vectors were grown on LB Kan medium.

### *In vitro *LR recombination

LR recombination reactions were performed using the Gateway LR Clonase enzyme mix (Invitrogen) as described in the manufacturer's protocol. Modifications are described. Briefly, the enzyme mix contains the bacteriophage lambda recombination proteins Integrase (Int) and Excisionase (Xis), and the *E. coli*-encoded protein Integration Host Factor (IHF). Each reaction was performed in a 20 μl volume, using 150 ng of DEST-BAC DNA and 100 ng of entry vector DNA. Reactions were incubated overnight at room temperature. After Proteinase K treatment, ten-fold and 100-fold dilutions were transformed into electrocompetent DH10B cells (Invitrogen). The electroporation conditions employed 0.1 cm cuvets and 1.7 kV/25 μF/200 Ω settings on a BioRad GenePulser II. Clones were confirmed by PCR with primer pairs targeting sequences in the inserted genes (DsRed2, A33R, B5R), and with HSV-2 *UL41*-specific primers (UL_41_F; 5'GGGGGTCTTCTTCGTAGTCG and UL_41_R; 5'ACATCAGCACCGGCTACATT) hybridizing close to the site of insertion whereby generating fragments of different size according to the inserted gene. A primer pair that amplifies a fragment in the *EGFP *gene was used to show loss of *EGFP *in recombinant clones.

### BAC mutagenesis

Mutagenesis of HSV2-BAC was performed as described previously, employing a BAC replacement vector containing a temperature-sensitive origin of replication and marker genes for positive and negative selection during the BAC mutagenesis procedure [4,15]. Briefly, electrocompetent RR1 *E. coli *cells were transformed with HSV2-BAC DNA and selected for chloramphenicol resistance. The electroporation conditions employed 0.1 cm cuvets and 1.7 kV/25 μF/200 Ω settings on a BioRad GenePulser II. Clones were confirmed by PCR with 3 independent HSV-2 specific primer pairs and a primer pair that amplifies a fragment in the chloramphenicol acetyltransferase (*cat*) gene. A 620 bp fragment in the *cat *gene was amplified with the following primers: MM507 (5'GCCCATGGTGAAAACGGGGGC) and MM508 (5'GATCGGCACGTAAGAGGTTCC).

Electrocompetent RR1 cells harboring the HSV2-BAC were prepared as previously described [29], and were electroporated with 10 ng of the respective pKO5.2-derived shuttle vector (pKO5.2-C.1-gD-EGFP or pKO5.2-UL41 *att*R-EGFP), and plated onto chloramphenicol/zeocin (Cam/Zeo) plates in serial dilutions at 30°C. The following day, multiple colonies were picked into 1 ml of LB broth and plated in serial dilutions onto Cam/Zeo plates and incubated at 43°C. Colonies at 43°C were analyzed by picking several into 1 ml LB each and plating out 20 μl onto sucrose/chloramphenicol (Suc/Cam) plates at 30°C. Approximately 10 colonies from each plate, representing an original 43°C colony, were transferred to grids on Suc/Zeo and Cam LB plates at 30°C. Colonies that were Cam+, Zeo/Suc- were picked into 100 μl of 10 mM Tris pH 8.0 for colony PCR using HSV-2 specific primers. along with either a primer pair flanking the *UL41 *coding region (for insertion of the *att*R-*EGFP *cassette) or a primer pair flanking the *gD *coding region (for insertion of *EGFP *to delete *US6*). Positive colonies were streaked on Cam plates and grown at 37°C. Large scale BAC vector DNA isolation was performed using the Large Construct kit (Qiagen Inc., Valencia, CA).

### Cells and viruses

Vero cells were obtained from the American Type Culture Collection (ATCC, Manassas, VA) and maintained in Dulbecco's Minimal Essential Medium (DMEM) supplemented with 10% fetal bovine serum, FBS (HyClone, Logan, UT); 2 mM Glutamax-1; and 0.05 mg/ml Gentamicin (Gibco/Invitrogen, Grand Island, NY). VD60 cells, a Vero cell line that expresses the HSV-1 gD [30], were a gift from Dr. David C. Johnson, Department of Molecular Microbiology & Immunology, Oregon Health Sciences University. VD60 cells were maintained as suggested in Eagle MEM lacking histidine (MEM-his) supplemented with 1 mM histidinol (Sigma Chemical Co., St. Louis, MO) and 5% FBS. Prior to infection, VD60 cells were passaged two times in DMEM containing 10% FBS. Cells were seeded in 6-well plates at 8 × 10^5^/well one day before infection or transfection. The HSV-2 (MS) BAC used as the parental clone for subsequent recombinant BACs described in this study has been described previously [17].

### Transfection of BAC DNA

Vero cells were transfected using Lipofectamine (Invitrogen, Carlsbad, CA), at a ratio of five μl per μg of plasmid or BAC DNA. At 5 h after transfection, an equal volume of complete media was added; media was changed again the next day. Infected cell lysates were harvested and used to infect Vero cells seeded at 2 × 10^6^/flask in 25 cm^2 ^tissue culture vessels.

### Infection of cells

For Western blot, cells were infected at a multiplicity of infection (moi) of 5; for immunostaining, virus stocks were titered to get a sufficient number of separated plaques per well. Virus was added to the cell sheet in about 1 ml of complete media per well. Plates were incubated at 37°C for 2 hours, with gentle mixing of the media by shaking the plate every 15 minutes. After that, cells were overlaid with complete media (Western blot) or methylcellulose (immunostaining).

### Immunostaining of plaques

Infected cell sheets were fixed using a 1:1 mixture of methanol/acetone. All wash steps were performed using Tris buffered saline (TBS) with 0.1% Triton-X. Fetal bovine serum at a concentration of 20% in TBS was used for blocking. Mouse anti-HA monoclonal antibody was used as primary antibody (diluted 1:1000). Detection was performed with goat anti-mouse polyclonal antibodies conjugated to alkaline phosphatase (Promega, Madison, WI) in combination with Western Blue stabilized substrate for alkaline phosphatase (Promega). Pictures were taken with a Canon G3 digital camera equipped with a HOYA +4 macro lens.

### Western blot

Two days after infection, infected cells were harvested and resuspended in lysis Buffer (50 mM Tris Cl pH 7.5; 150 mM NaCl; 1% Triton X-100) with EDTA-free Protease Inhibitor Cocktail Tablets (Roche, Mannheim, Germany). Proteins were fractionated on precast NuPAGE 12% Bis-Tris gels (Invitrogen, Carlsbad, CA) and transferred electrophoretically onto PVDF membrane (Bio-Rad Laboratories, Hercules, CA) for subsequent Western blot. Mouse anti-HA monoclonal antibody (Covance, Richmond, CA) at a dilution of 1:1000 was used to detect HA-tagged A33R and B5R. Protein bands were detected by chemiluminescence, using horseradish peroxidase conjugated sheep anti-mouse antibody (Amersham Biosciences, Piscataway, NJ) at a dilution of 1:30000, and SuperSignal West Dura detection reagents (Pierce, Rockford, IL). Digital pictures were captured with a Fuji LAS-3000 CCD camera and software supplied by the manufacturer.

## Authors' contributions

FS participated in the design of the study, carried out the molecular biology studies and helped to draft the manuscript. JPW participated in the design of the study and its coordination and drafted the manuscript. Both authors read and approved the final manuscript.
